# Supplementary material for: No Association between Genetic Variants of the COMT and OPRM1 Genes and Pain Perception among Patients Undergoing Total Hip or Knee Arthroplasty for Primary Osteoarthritis
Source: Genes (Basel). 2022 Oct 1;13(10):1775. doi: 10.3390/genes13101775 (PMC9602431; doi:10.3390/genes13101775)
Supplement: Supplementary file 1 [file genes-13-01775-s001.zip › genes-1882311-supplementary.pdf]

Table S1. Association of the *COMT* rs4633 with pain tolerance and pain threshold.

Pain characteristic	General			Dominant		Recessive	
	**TT (*n* = 61)**	**CT (*n* = 92)**	**CC (*n* = 50)**	**CC+CT (*n* = 142)**	**TT (*n* = 61)**	**CC (*n* = 50)**	**CT+TT (*n* = 153)**
Pain threshold (LH)	6.71 ± 2.32	6.05 ± 2.41	6.30 ± 2.43	6.14 ± 2.41	6.71 ± 2.32	6.30 ± 2.43	6.31 ± 2.39
		0.327/0.657		0.196/0.606		0.975/0.975	
Pain threshold (RH)	7.95 ± 2.45	6.83 ± 2.65	6.96 ± 2.60	6.87 ± 2.62	7.95 ± 2.45	6.96 ± 2.60	7.28 ± 2.62
		**0.041**/0.246		**0.012**/0.246		**0.467**/0.657	
Pain threshold (VASLH)	4.30 ± 1.88	3.73 ± 1.58	3.82 ± 1.76	3.76 ± 1.64	4.30 ± 1.88	3.82 ± 1.76	3.95 ± 1.72
		0.079/0.3792		**0.024**/0.246		**0.445**/0.657	
Pain threshold (VASRH)	4.26 ± 1.85	3.71 ± 1.73	3.88 ± 1.98	3.77 ± 1.81	4.26 ± 1.85	3.88 ± 1.98	3.93 ± 1.79
		0.118/0.472		**0.040**/0.246		0.628/0.753	
Pain tolerance (LH)	11.30 ± 4.09	10.60 ± 4.0	10.60 ± 4.28	10.60 ± 4.09	11.30 ± 4.09	10.60 ± 4.28	10.90 ± 4.04
		0.862/0.899		0.589/0.744		0.782/0.853	
Pain tolerance (RH)	11.90 ± 4.07	10.80 ± 3.90	11.40 ± 4.97	11.00 ± 4.30	11.90 ± 4.07	11.40 ± 4.97	11.20 ± 3.99
		0.487/0.657		0.339/0.657		0.750/0.853	
Pain tolerance (VASLH)	7.03 ± 1.71	6.68 ± 1.68	6.69 ± 1.84	6.68 ± 1.73	7.03 ± 1.71	6.69 ± 1.84	6.82 ± 1.70
		0.433/0.657		0.202/0.606		0.493/0.657	
Pain tolerance (VASRH)	7.33 ± 1.40	7.08 ± 1.64	7.07 ± 1.67	7.07 ± 1.64	7.33 ± 1.40	7.07 ± 1.67	7.18 ± 1.55
		0.454/0.657		0.240/0.640		0.394/0.657	

Adjusted for age and sex. LH- left hand, RH-right hand, VAS – visual analog scale. After slash – FDR adjusted P values

Table S2. Association of the *COMT* rs4680 with pain tolerance and pain threshold.

Pain characteristic	General			Dominant		Recessive	
	**AA (*n* = 61)**	**AG (*n* = 93)**	**GG (*n* = 49)**	**GG+AG (*n* = 142)**	**AA (*n* = 61)**	**GG (*n* = 49)**	**AG+AA (*n* = 154)**
Pain threshold (LH)	6.71 ± 2.32	6.04 ± 2.40	6.32 ± 2.45	6.14 ± 2.41	6.71 ± 2.32	6.32 ± 2.45	6.31 ± 2.38
		0.368/0.657		0.196/0.606		0.953/0.953	
Pain threshold (RH)	7.95 ± 2.45	6.81 ± 2.64	6.99 ± 2.61	6.87 ± 2.62	7.95 ± 2.45	6.99 ± 2.61	7.26 ± 2.62
		**0.040**/0.240		**0.012**/0.240		0.522/0.659	
Pain threshold (VASLH)	4.30 ± 1.88	3.74 ± 1.57	3.80 ± 1.77	3.76 ± 1.64	4.30 ± 1.88	3.80 ± 1.77	3.96 ± 1.72
		0.079/0.379		**0.024**/0.240		0.385/0.657	
Pain threshold (VASRH)	4.26 ± 1.85	3.73 ± 1.73	3.84 ± 1.97	3.77 ± 1.81	4.26 ± 1.85	3.84 ± 1.97	3.94 ± 1.79
		0.123/0.492		**0.040**/0.240		0.503/0.659	
Pain tolerance (LH)	11.30 ± 4.09	10.60 ± 3.99	10.70 ± 4.32	10.60 ± 4.09	11.30 ± 4.09	10.70 ± 4.32	10.90 ± 4.03
		0.863/0.900		0.589/0.706		0.806/0.879	
Pain tolerance (RH)	11.90 ± 4.07	10.80 ± 3.89	11.50 ± 5.00	11.00 ± 4.30	11.90 ± 4.07	11.50 ± 5.00	11.20 ± 3.98
		0.453/0.657		0.339/0.657		0.682/0.779	
Pain tolerance (VASLH)	7.03 ± 1.71	6.68 ± 1.67	6.68 ± 1.86	6.68 ± 1.73	7.03 ± 1.71	6.68 ± 1.86	6.82 ± 1.69
		0.428/0.657		0.202/0.606		0.466/0.657	
Pain tolerance (VASRH)	7.33 ± 1.40	7.09 ± 1.63	7.05 ± 1.68	7.07 ± 1.64	7.33 ± 1.40	7.05 ± 1.68	7.18 ± 1.54
		0.426/0.657		0.240/0.640		0.335/0.657	

Adjusted for age and sex. LH-left hand, RH-right hand, VAS – visual analog scale; PPT: pressure pain threshold, PTOL: pressure pain tolerance. After slash – FDR adjusted P values

Table S3. Association of the *COMT* rs4818 with pain tolerance and pain threshold.

Pain characteristic	General			Dominant		Recessive	
	**CC (*n* = 82)**	**CG (*n* = 98)**	**GG (*n* = 23)**	**GG+CG (*n* = 121)**	**CC (*n* = 82)**	**GG (*n* = 23)**	**CG+CC (*n* = 180)**
Pain threshold (LH)	6.57 ± 2.26	6.07 ± 2.58	6.40 ± 2.00	6.13 ± 2.47	6.57 ± 2.26	6.40 ± 2.00	6.30 ± 2.44
		0.561/0.674		0.347/0.520		0.819/0.849	
Pain threshold (RH)	7.78 ± 2.47	6.73 ± 2.77	7.09 ± 2.02	6.80 ± 2.64	7.78 ± 2.47	7.09 ± 2.02	7.21 ± 2.69
		**0.048**/0.392		**0.016**/0.384		0.849/0.849	
Pain threshold (VASLH)	4.16 ± 1.80	3.79 ± 1.64	3.65 ± 1.80	3.76 ± 1.66	4.16 ± 1.80	3.65 ± 1.80	3.96 ± 1.72
		0.134/0.520		**0.049**/0.392		0.347/0.520	
Pain threshold (VASRH)	4.12 ± 1.81	3.84 ± 1.79	3.52 ± 2.09	3.78 ± 1.84	4.12 ± 1.81	3.52 ± 2.09	3.97 ± 1.80
		0.158/0.520		0.081/0.486		0.203/0.520	
Pain tolerance (LH)	11.20 ± 4.12	10.40 ± 4.05	11.20 ± 4.14	10.60 ± 4.06	11.20 ± 4.12	11.20 ± 4.14	10.80 ± 4.09
		0.659/0.718		0.590/0.674		0.581/0.674	
Pain tolerance (RH)	11.70 ± 3.99	10.70 ± 4.27	12.20 ± 4.75	11.00 ± 4.39	11.70 ± 3.99	12.20 ± 4.75	11.10 ± 4.17
		0.243/0.520		0.434/0.612		0.231/0.520	
Pain tolerance (VASLH)	6.94 ± 1.64	6.73 ± 1.82	6.50 ± 1.67	6.69 ± 1.79	6.94 ± 1.64	6.50 ± 1.67	6.83 ± 1.74
		0.467/0.622		0.307/0.520		0.337/0.520	
Pain tolerance (VASRH)	7.29 ± 1.43	7.10 ± 1.63	6.85 ± 1.84	7.05 ± 1.67	7.29 ± 1.43	6.85 ± 1.84	7.19 ± 1.54
		0.286/0.520		0.184/0.520		0.228/0.520	

Adjusted for age and sex. LH- left hand, RH-right hand, VAS – visual analog scale; PPT: pressure pain threshold, PTOL: pressure pain tolerance. After slash – FDR adjusted P values

Table S4. Association of the *COMT* rs6269 with pain tolerance and pain threshold.

Pain characteristic	General			Dominant		Recessive	
	**AA (*n* = 82)**	**GA (*n* = 98)**	**GG (*n* = 23)**	**GG+GA (*n* = 121)**	**AA (*n* = 82)**	**GG (*n* = 23)**	**GA+AA (*n* = 180)**
Pain threshold (LH)	6.57 ± 2.26	6.06 ± 2.58	6.44 ± 1.97	6.13 ± 2.74	6.57 ± 2.26	6.44 ± 1.97	6.29 ± 2.45
		0.499/0.700		0.347/0.682		0.680/0.709	
Pain threshold (RH)	7.78 ± 2.47	6.75 ± 2.77	7.01 ± 2.05	6.80 ± 2.64	7.78 ± 2.47	7.01 ± 2.05	7.22 ± 2.68
		**0.050**/0.400		**0.016**/0.384		0.775/0.775	
Pain threshold (VASLH)	4.16 ± 1.80	3.76 ± 1.64	3.78 ± 1.78	3.76 ± 1.66	4.16 ± 1.80	3.78 ± 1.78	3.94 ± 1.72
		0.145/0.544		**0.049**/0.400		0.579/0.700	
Pain threshold (VASLH)	4.12 ± 1.81	3.80 ± 1.78	3.70 ± 2.12	3.78 ± 1.84	4.12 ± 1.81	3.70 ± 2.12	3.94 ± 1.80
		0.209/0.544		0.081/0.486		0.428/0.682	
Pain tolerance (LH)	11.20 ± 4.12	10.40 ± 4.06	11.20 ± 4.12	10.60 ± 4.06	11.20 ± 4.12	11.20 ± 4.12	10.80 ± 4.10
		0.556/0.700		0.590/0.700		0.455/0.682	
Pain tolerance (RH)	11.70 ± 3.99	10.70 ± 4.27	12.20 ± 4.73	11.00 ± 4.39	11.70 ± 3.99	12.20 ± 4.73	11.10 ± 4.17
		0.189/0.544		0.434/0.682		0.176/0.544	
Pain tolerance (VASLH)	6.94 ± 1.64	6.70 ± 1.84	6.63 ± 1.58	6.69 ± 1.79	6.94 ± 1.64	6.63 ± 1.58	6.81 ± 1.75
		0.227/0.544		0.307/0.669		0.613/0.700	
Pain tolerance (VASRH)	7.29 ± 1.43	7.05 ± 1.68	7.07 ± 1.65	7.05 ± 1.67	7.29 ± 1.34	7.07 ± 1.65	7.16 ± 1.57
		0.414/0.682		0.184/0.544		0.671/0.709	

Adjusted for age and sex. LH- left hand, RH-right hand, VAS – visual analog scale; PPT: pressure pain threshold, PTOL: pressure pain tolerance. After slash – FDR adjusted P values

Table S5. Association of the *OPRM1* rs1799971 with pain tolerance and pain threshold.

Pain characteristic	General			Dominant		Recessive	
	**AA (*n* = 163)**	**AG (*n* = 36)**	**GG (*n* = 3)**	**GG+AG (*n* = 39)**	**AA (*n* = 163)**	**GG (*n* = 3)**	**AG+AA (*n* = 199)**
Pain threshold (LH)	6.34 ± 2.50	6.09 ± 1.88	5.63 ± 1.00	6.06 ± 1.82	6.34 ± 2.50		
		0.848/0.878		0.566/0.754		NA	
Pain threshold (RH)	7.14 ± 2.68	7.38 ± 2.37	6.77 ± 0.81	7.33 ± 2.29	7.14 ± 2.68		
		0.878/0.878		0.628/0.772		NA	
Pain threshold (VASLH)	3.87 ± 1.73	3.97 ± 1.59	6.67 ± 1.53	4.18 ± 1.73	3.87 ± 1.73		
		**0.026**/0.208		0.310/0.551		NA	
Pain threshold (VASRH)	3.82 ± 1.82	4.17 ± 1.66	7.00 ± 1.73	4.38 ± 1.82	3.82 ± 1.82		
		**0.011**/0.176		0.081/0.324		NA	
Pain tolerance (LH)	11.00 ± 4.27	10.30 ± 3.26	7.67 ± 0.90	10.10 ± 3.22	11.00 ± 4.27		
		0.450/0.720		0.256/0.512		NA	
Pain tolerance (RH)	11.30 ± 4.26	10.90 ± 4.09	9.37 ± 1.31	10.80 ± 3.96	11.30 ± 4.26		
		0.809/0.878		0.526/0.754		NA	
Pain tolerance (VASLH)	6.70 ± 1.72	7.03 ± 1.75	8.67 ± 1.15	7.15 ± 1.76	6.70 ± 1.72		
		0.068/0.324		0.119/0.368		NA	
Pain tolerance (VASRH)	7.08 ± 1.53	7.33 ± 1.79	8.67 ± 0.58	7.44 ± 1.76	7.08 ± 1.53		
		0.138/0.368		0.171/0.390		NA	

Adjusted for age and sex. LH- left hand, RH-right hand, VAS – visual analog scale; PPT: pressure pain threshold, PTOL: pressure pain tolerance. After slash – FDR adjusted P values
